# Complete Genome Sequence of Pseudomonas stutzeri Strain F2a, Isolated from Seleniferous Soil

**DOI:** 10.1128/MRA.00631-21

**Published:** 2021-08-19

**Authors:** Masao Inoue, Yuu Hirose, Ryuta Tobe, Shigeki Saito, Riku Aono, N. Tejo Prakash, Hisaaki Mihara

**Affiliations:** a College of Life Sciences, Ritsumeikan University, Kusatsu, Shiga, Japan; b R-GIRO, Ritsumeikan University, Kusatsu, Shiga, Japan; c Department of Applied Chemistry and Life Science, Toyohashi University of Technology, Toyohashi, Aichi, Japan; d School of Energy and Environment, Thapar Institute of Engineering and Technology, Patiala, Punjab, India; University of Arizona

## Abstract

Pseudomonas stutzeri is a potential candidate for bioremediation of selenium-contaminated grounds and waters. Here, we report the complete genome sequence of a novel strain, F2a, which was isolated from a seleniferous area of Punjab, India. The genome sequence provides insight into the potential selenium oxyanion-reducing activity of this strain.

## ANNOUNCEMENT

Pseudomonas stutzeri is a Gram-negative, mesophilic, facultatively anaerobic bacterium with some strains showing reducing activity to selenium and tellurium oxyanions (1–4). Thus, this species is considered a potential candidate for bioremediation of contaminated grounds and waters. Here, we report the complete genome sequence of a novel strain, F2a, which was isolated from selenium-contaminated soil.

A soil sample was collected from the rhizosphere (within a 1-cm radius of a plant root and 10 cm below the soil surface) of a wheat crop in a seleniferous belt bordering the Nawanshahr-Hoshiarpur region in Punjab State, India (31°56′N 75°55′E) (5, 6). A spatula of the soil sample was suspended in a tryptic soy broth (TSB) liquid medium, and a portion of the suspension was streaked onto a TSB agar medium supplemented with 10 mM sodium selenite. A single colony of the strain F2a was isolated aerobically at 30°C. For genomic DNA extraction, the isolated cells were aerobically grown in TSB. Genomic DNA extraction, library preparation, sequencing, and assembly were performed as described previously (7). An 800-bp paired-end library and an 8-kbp mate-pair library were prepared using the TruSeq DNA PCR-free sample preparation kit (Illumina, San Diego, CA) and Nextera mate-pair sample preparation kit (Illumina), respectively. Sequencing was performed on an Illumina MiSeq instrument with MiSeq reagent kit v3 (600 cycles), which generated 1.57 million paired-end reads (0.94 Gbp) and 2.57 million mate-pair reads (1.36 Gbp). Correction of sequence errors based on a 17-mer frequency and removal of junction sequences of the mate-pair reads were performed using ShortReadManager (8). The processed reads were assembled using Newbler v2.8 (Roche Diagnostics, Indianapolis, IN, USA), yielding a circular scaffold consisting of 21 contigs (>0.5 kbp) with 208× coverage of the genome of the strain F2a. The sequence gaps between the contigs in the scaffold were determined *in silico* using GenoFinisher and AceFileViewer as described previously (8), which visualize the connection of unplaced repeat sequences based on the Ace file of the Newbler output (http://www.ige.tohoku.ac.jp/joho/gf/index.php). Annotation was performed with the DFAST server v1.4.0 (9). Genomic comparison was performed using OAT v0.93.1 (10) and GGDC v2.1 (11). A homology search for 16S rRNA gene sequences and protein amino acid sequences was carried out using BLAST (12). Default parameters were used for all software unless otherwise noted.

We succeeded in determining the complete genome sequence of the strain F2a, which comprises a circular chromosome, a total length of 4,545,686 bp, and an average G+C content of 64.3%. The numbers of predicted protein-coding genes, rRNAs, and tRNAs were 4,197, 12, and 66, respectively. The average nucleotide identity, digital DNA-DNA hybridization, and sequence identities of 16S rRNA genes between genomes of the strain F2a and P. stutzeri strain ATCC 17588^T^ (= CGMCC 1.1803) (RefSeq assembly accession number GCF_000219605.1) were 97.96%, 82.20%, and 99.87% to 99.93%, respectively. These results suggest that strain F2a is a member of the species P. stutzeri, while the genome is slightly different from that of the type strain.

We identified a putative glutathione reductase gene (PszF2a_21280) in the F2a genome, which is essential for the selenite-reducing activity of P. stutzeri (13), with a sequence identity of 98.89%. Moreover, the strain F2a harbors several glutathione-, glutaredoxin-, and thioredoxin-like genes, as does the type strain of P. stutzeri. These results were consistent with the fact that strain F2a was isolated from a selenium-rich environment, implying its ability to detoxify selenium oxyanions.

### Data availability.

The complete genome sequence was deposited in GenBank under accession number AP024722.1. The raw reads for mate-pair and paired-end sequencing were deposited in SRA under accession numbers DRR299385 and DRR299386, respectively.
